# Role and regulation of Yap in KrasG12D-induced lung cancer

**DOI:** 10.18632/oncotarget.22865

**Published:** 2017-12-02

**Authors:** Yaopan Mao, Shuguo Sun, Kenneth D. Irvine

**Affiliations:** ^1^ Waksman Institute, Cancer Institute of New Jersey, Department of Molecular Biology and Biochemistry, Rutgers University, Piscataway, NJ 08854, USA; ^2^ Current address: Department of Anatomy, Tongji Medical College, Huazhong University of Science and Technology, Wuhan 430030, China

**Keywords:** YAP, Kras, adenocarcinoma, lung cancer

## Abstract

The Hippo pathway and its downstream transcriptional co-activator Yap influence lung cancer, but the nature of the Yap contribution has been unclear. Using a genetically engineered mouse lung cancer model, we show that Yap deletion completely blocks KrasG12D and p53 loss-driven adenocarcinoma initiation and progression, whereas heterozygosity for Yap partially suppresses lung cancer growth and progression. We also characterize Yap expression during tumor progression and find that nuclear Yap can be detected from the earliest stages of lung carcinogenesis, but at levels comparable to that in aveolar type II cells, which are a cell of origin for lung adenocarcinoma. At later stages of tumorigenesis, variations in Yap levels are detected, which correlate with differences in cell proliferation within tumors. Our observations imply that Yap is not directly activated by oncogenic Kras during lung tumorigenesis, but is nonetheless absolutely required for this tumorigenesis, and support Yap as a therapeutic target in lung adenocarcinoma.

## INTRODUCTION

Lung cancer is the leading cause of cancer deaths, both in the United States and world-wide [1]. Activating mutations in EGFR, or one of its downstream effectors, KRAS, are amongst the most common genetic lesions associated with adenocarcinoma, which comprises approximately 40% of lung cancers [1–3]. Additionally, mutations in p53, one of the most frequently mutated tumor suppressor genes in human cancers, have been observed in close to half of adenocarcinomas, often in conjunction with activating mutations in EGFR or KRAS [4]. Tyrosine kinase inhibitors can be used to treat lung cancers associated with EGFR mutations, but tumors eventually become resistant, and effective treatments for KRAS-induced cancers are currently lacking. Better delineation of genetic programs essential for lung tumorigenesis could help identify therapeutic targets. Here, we describe investigations of the requirements and expression of Yap in murine lung tumors induced by expression of activated-Kras and deletion of p53.

**Figure 1:**
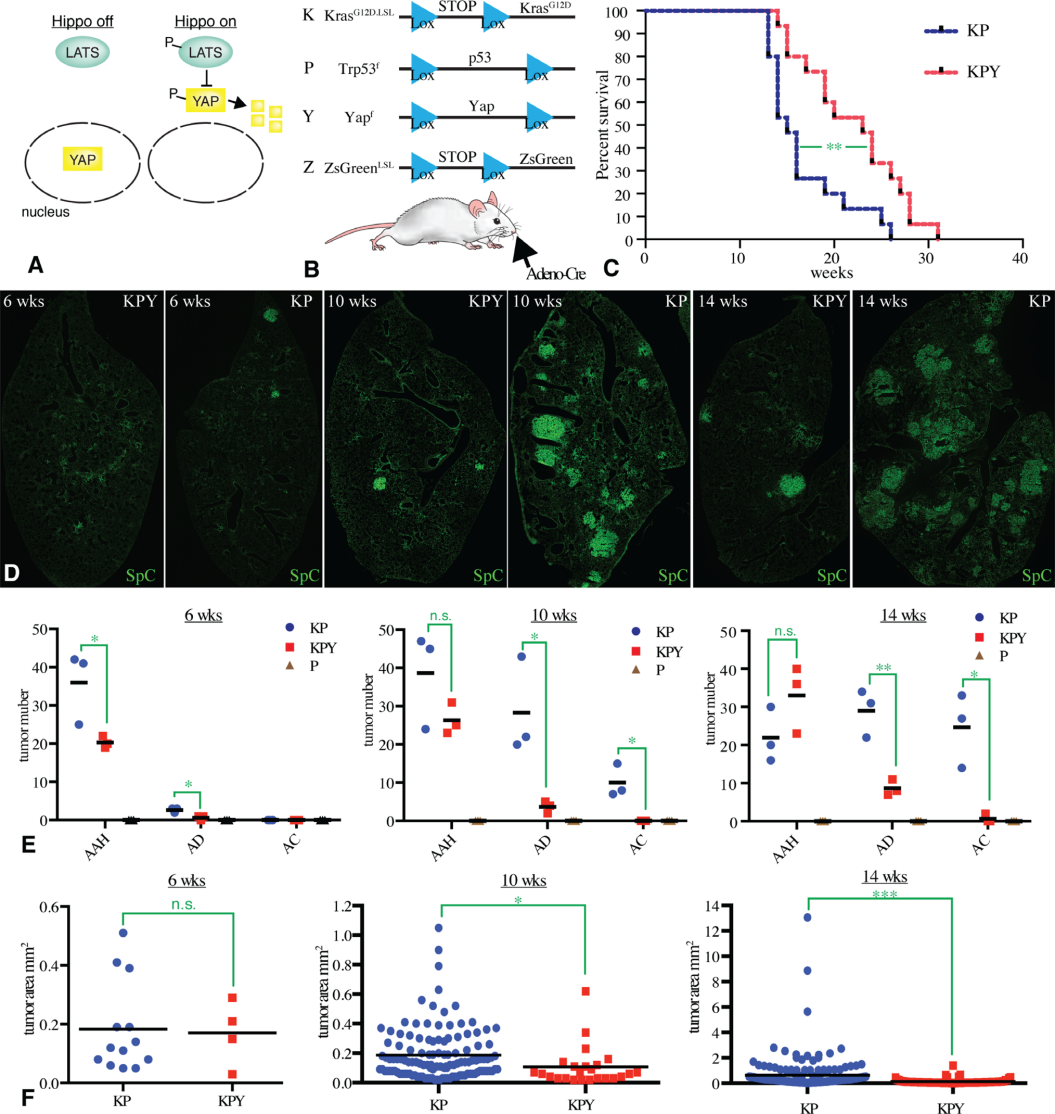
YAP contributes to Kras-driven tumorigenesis (**A**) Simple Hippo pathway schematic. When Hippo pathway activity is low, Lats kinases are inactive, and Yap can accumulate in the nucleus. Activation of the Hippo pathway promotes activation of Lats kinases, which phosphorylate Yap. This phosphorylation promotes exclusion of Yap from the nucleus, and Yap degradation. (**B**) Schematic illustration of the experimental design and alleles used in this study. Intranasal inhalation of Cre-expressing adenovirus triggers recombination at Lox sites (blue triangles), leading to expression of Kras^G12D^ or ZsGreen, and excision of Yap or p53. (**C**) Survival of 15 KP and 15 KPY male mice, infected at 6–8 weeks of age, in weeks after infection,. Statistical comparison by log-rank test, *P* value = 0.005 (^**^). (**D**) Representative tiled confocal scans of frozen sections of individual lung lobes from KP or KPY mice, as indicated, at 6, 10, or 14 weeks after infections. Staining for SpC (green) is shown to indicate tumor number and size. (**E**) Classifications of tumor numbers by scatter plot from KP, KPY, and P (negative control) mice at 6, 10, and 14 weeks after infection, with three mice analyzed per genotype, and mean values indicated by the black bars. Significance of differences between KP and KPY mice indicated by unpaired *t*-test, n.s. indicates *P* > 0.05, ^*^indicates *P* < 0.05, ^**^indicates *P* < 0.01. (**F**) Classifications of tumor area by scatter plot from KP and KPY mice at 6, 10, and 14 weeks after infection, with three mice analyzed per genotype, and mean values indicated by the black bars, and statistical comparisons by unpaired *t*-test, n.s. indicates *P >* 0.05, ^*^indicates *P* < 0.05, ^***^indicates *P* < 0.001.

Yap and its orthologue Taz are transcription factors regulated by Hippo signaling. Hippo signaling is a conserved signal transduction network, first identified in *Drosophila*, which plays essential roles in regulating organ growth and cell fate [5–7]. Hippo signaling reduces growth by inhibiting Yap and Taz. [8, 9]. This occurs principally through phosphorylation of Yap and Taz by Lats family kinases (Lats1 and Lats2), which decreases their nuclear localization and promotes their degradation. Thus, when Hippo pathway activity is low, levels of Yap and Taz proteins, and nuclear localization of Yap and Taz, are increased (Figure 1A). Yap and Taz can also be regulated by other kinases that are not affected by the Hippo pathway, including Src and AMPK, and also by kinase-independent sequestration mechanisms [5, 9, 10].

Elevated levels and nuclear localization of Yap and Taz have been reported in a wide range of cancers, including colorectal, lung, breast, gastric, and ovarian cancers [7, 11, 12]. For example, examination of non-small cell lung cancer (NSCLC), which includes adenocarcinoma, has revealed that roughly 70% of cases are associated with visibly elevated levels and/or nuclear localization of YAP or TAZ, and that elevated expression of YAP/TAZ correlates with poor prognosis [12–18]. However, in most cases the mechanisms responsible for YAP/TAZ activation in human tumors, and the contribution of this YAP/TAZ activation to tumorigenesis, have not been clearly defined. Moreover, mutations in what are considered core upstream tumor suppressors of the Hippo pathway have only rarely been observed in most human cancers, raising the question of what is responsible for the elevated levels of YAP/TAZ. One potential answer is dysregulation of other pathways that cross-talk with Hippo signaling. Extensive cross talk between Hippo and other signaling pathways has been reported [19], including with EGFR signaling. Activation of EGFR and/or RAS can promotes YAP activation in mammalian cell culture models and in *Drosophila*, and at least three different mechanisms by which it can do so have been described, including inhibiting LATS kinases [20, 21], and LATS-independent regulation of YAP stability [22].

The relationship between EGFR-RAS activity and YAP/TAZ activity in lung cancer has been examined, but nonetheless there remain substantial gaps in our understanding of the relationship between them during lung cancer progression. Studies in human cell lines or in mouse models reported a contribution of Yap to Kras-induced tumorigenesis, while implying, but not confirming, that tumors could still form in the absence of Yap [23, 24]. Elevated levels of Yap have been reported both in human NSCLC and in mouse models of Kras-induced NSCLC, but published studies have varied in their description of Yap levels and localization induced by Kras. It has also been suggested that Yap levels and nuclear localization are increased by tumor-promoting mutations, including p53 or Lkb1 [13, 14, 23, 24].

YAP and TAZ also have roles in normal lung, both during lung development, and in maintaining lung homeostasis and recovery from injury [25–28]. Notably, Yap acts in progenitor cells required for lung development and homeostasis, including aveolar type 2 (AT2) cells, and contributes to maintenance of progenitor cell fates [28]. Although there has been debate about cell types of origin for adenocarcinoma, the AT2 cells have been clearly implicated as a source of adenocarcinomas both in human cancers and in mouse models, including *Kras*^*G12D*^-induced NSCLC [29–31]. In adult lungs these are progenitor cells for the more abundant type I aveolar cells, and their proliferation can be stimulated by lung injuries in order to maintain lung homeostasis.

Here, we describe our investigations of the role of Yap in a mouse lung cancer model induced by activated Kras and loss of p53 [32, 33]. We find that Yap is absolutely essential for this Kras-induced tumorigenesis. Nonetheless, quantitation of Yap levels reveals that Yap levels are not elevated by activation of Kras and loss of p53 during initial stages of tumorigenesis. As tumors progress Yap levels become heterogeneous, and elevated in a subset of cells, and Yap levels correlate with markers of cell proliferation. Our observations confirm an essential role for Yap in Kras-induced lung adenocarcinoma, and imply that the local environment modulates Yap activity during tumor progression.

## RESULTS

### Suppression of Kras-induced tumorigenesis by a conditional Yap allele

To evaluate requirements for Yap in lung tumors induced by activated-Kras, we employed a mouse model for lung adenocarcinoma in which expression of an oncogenic (activated) allele of Kras (Kras^G12D^) is dependent upon Cre recombinase-mediated excision of a transcriptional terminator from an inactive allele (Kras^G12D.LSL^), initiated by inhalation of an adenovirus-expressing Cre recombinase [33] (Figure 1B). The resulting Kras^G12D^-expressing cells readily form hyperplasias or adenomas, but only slowly and less frequently adenocarcinomas. However, if expression of Kras^G12D^ is coupled to loss of p53, by including a conditional *Trp53* allele (*Trp53*^*f*^), then highly proliferative adenocarcinomas form frequently and rapidly [32]. The *Kras*^*G12D*^ *Trp53*^*-*^ model has been well-characterized and replicates features of human lung adenocarcinoma, including histology, pathology, and gene expression profile [23, 32, 34]. The requirement for Yap in this model was assessed by including a conditional allele of Yap (*Yap*^*f*^), and comparing *Kras*^*G12D.LSL*^*; p53*^*f/f*^ (abbreviated to KP) mice to *Kras*^*G12D.LSL*^*; p53*^*f/f*^ *Yap*^*f/f*^ (KPY) mice (Figure 1B). As an initial comparison to investigate the contribution of Yap to tumorigenesis, we examined differences in survival between KP mice and KPY mice. The mean survival time of KPY mice was about 50% longer after infection with Adeno-Cre than the mean survival of KP mice (Figure 1C, Log-rank test, *P* value = 0.005, 15 male mice for each group). Autopsy revealed that in both genotypes all the animals that became moribund had lung tumors.

To directly compare tumors between KP and KPY mice, lung sections were stained with DAPI to label nuclei, and with antibodies recognizing Surfactant protein-C (SpC), E-cadherin, and Yap, and examined by confocal microscopy. SpC is expressed specifically in AT2 cells, which are the main cell of origin for Kras-induced lung adenocarcinoma [29–31]. Normal alveolar lung tissue is characterized by dispersed, isolated Sp-C positive cells, whereas Kras-induced lung tumors are characterized by clusters of Sp-C positive cells. Differences in tumor number, progression, and size were observed between KP and KPY mice when examined at 6, 10, and 14 weeks after infection with Adeno-Cre (Figure 1D–1F). Small, irregular clusters of Sp-C positive cells that don’t disrupt aveolar architecture were classified as atypical adenomatous hyperplasia (AAH), larger, cohesive clusters of Sp-C positive cells that disrupt aveolar architecture were classified as adenomas (AD), large clusters of Sp-C positive cells with irregular borders and/or heterogeneous SpC expression, and including cells with irregularly sized nuclei, were classified as adenocarcinomas (AC) [33]. In comparison to KP mice, KPY mice had substantially fewer tumors at all time points examined (Figure 1D, 1E). Tumor progression was also suppressed in KPY mice compared to KP mice, as they had many fewer adenomas and adenocarcinomas, but similar numbers of hyperplasias (Figure 1E). Also, hyperplasias and tumors tended to be smaller in KPY mice than in KP mice (Figure 1D, 1F).

### Yap is essential for Kras-induced tumorigenesis

**Figure 2:**
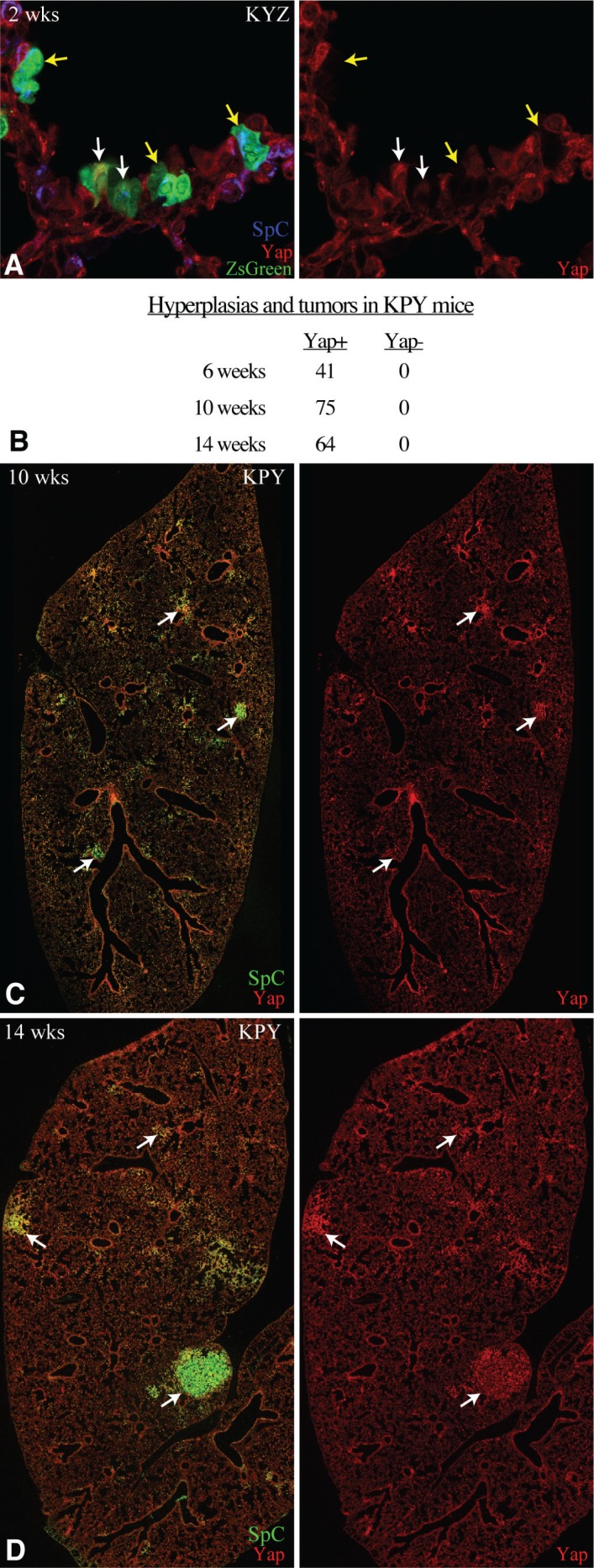
YAP is required for Kras-driven tumorigenesis (**A**) Representative confocal of lung section from KYZ mouse 2 weeks after infection with cells expressing ZsGreen, and stained for SpC (blue) and Yap (red). Examples of ZsGreen positive Yap negative cells are highlighted by yellow arrows, examples of ZsGreen positive Yap positive cells are highlighted by white arrows. (**B**) Quantitation of Yap positive and Yap negative AAH and tumors in three KPY mice. (**C**, **D**) Representative tiled confocal scans of frozen sections of lung lobes from KPY mice at 10 (C) or 14 (D) weeks after infections, stained for SpC (green) and Yap (red). White arrows highlight some examples of Yap-expressing tumors.

The comparison of tumorigensis between KP and KPY mice revealed a contribution of Yap to *Kras*^*G12D*^ *Trp53*^*–*^ -induced tumor progression, but interpretation of this contribution is complicated by the potential for Kras^G12D^ expression and *Trp53* excision to occur independently of *Yap* excision, as each depends on a separate Cre-mediated recombination event. The relative efficiency of Yap excision was estimated by examining the deletion frequency from Yap^f^ alleles in mice of genotype Kras^G12D.LSL^ Yap^f/f^ ZsGreen^LSL^ (KYZ) infected with Cre adenoviruses in the same way and at the same dosage as that of KP and KPY mice. The ZsGreen^LSL^ allele is mechanistically similar to the *Kras*^*G12D.LSL*^ allele, as it provides conditional expression of the fluorescent marker protein ZsGreen upon Cre recombinase-mediated excision of a floxed stop codon (Figure 1B). This positive marking by ZsGreen expression makes even single cells easy to identify. When lung sections from KYZ mice were examined by anti-Yap immunostaining and ZsGreen fluorescence two weeks after infection, 24 out of 50 green cells or small clones were Yap negative, as judges by anti-Yap staining (Figure 2A). These observations suggest that homozygous excision of both floxed Yap alleles happens in 48% of cells where the single recombination event needed to generate ZsGreen-expressing cells occurs. Assuming excision of each Yap^f^ allele occurs independently, excision of a single Yap^f^ allele would thus occur in about 70% of the cells where ZsGreen becomes expressed. Importantly, the absence of Yap staining in many ZsGreen-positive cells also confirmed the specificity of our anti-Yap immunostaining, and established that Yap is not required for viability of aveolar cells.

We then used Yap immunostaining, in conjunction with Sp-C, E-cadherin, and DNA staining, to examine Yap expression in hyperplasias and tumors generated by KP and KPY mice. Strikingly, out of >150 AAH, AD, and AC examined from KPY mice at 6, 10, and 14 weeks after viral infection, all expressed Yap protein (Figure 2B–2D). The complete absence of any Yap-negative tumors indicates that Yap is absolutely essential for the initiation of Kras^G12D^ p53^-^ lung tumorigenesis. The presence of Yap-expressing cells in tumors induced by Cre excisions within KPY mice presumably reflects the incomplete excision of the *Yap*^*f*^ allele, as was observed in KYZ mice. Indeed, the observation that overall tumors and hyperplasias occur in KPY mice at roughly half the frequency that they occur in KP mice (Figure 1D, 1E) would be consistent with recombination of *ZsGreen*^*LSL*^ and *Kras*^*G12D.LSL*^ alleles occurring at similar frequencies, such that roughly half of Kras^G12D^ expressing cells generated in KPY mice would be Yap negative, and hence unable to form tumors.

If Yap-negative cells completely fail to form tumors, and this accounts for the reduced tumor number, then what accounts for the delayed tumor progression and smaller tumor size observed in KPY mice as compared to KP mice? We suggest that it reflects a partial suppression of Kras-induced tumorigenesis by Yap heterozygosity. For example, if excision of each *Yap*^*f*^ allele occurred in 70% of cells with Kras^G12D^ expression, and homozygous *Yap* mutant cells fail to form hyperplasias or tumors, then one can calculate that over 82% of the Yap-expressing tumors in these animals would be expected to be heterozygous for the deleted and non-deleted *Yap*^*f*^ alleles. To directly examine this, we genotyped tumors from lung sections of KPY mice 14 weeks after infection, using laser capture micro dissection and PCR. Analysis of 10 tumors by this method revealed that they all had one copy of the original Yap^f^ allele and one copy of the deleted allele. These results confirm that Yap excision can be incomplete in KPY mice, and imply that growth and progression of Kras^G12D^ p53^-^ lung tumors is very sensitive to Yap levels.

### Yap levels are not elevated during initiation of Kras-induced tumorigenesis

**Figure 3:**
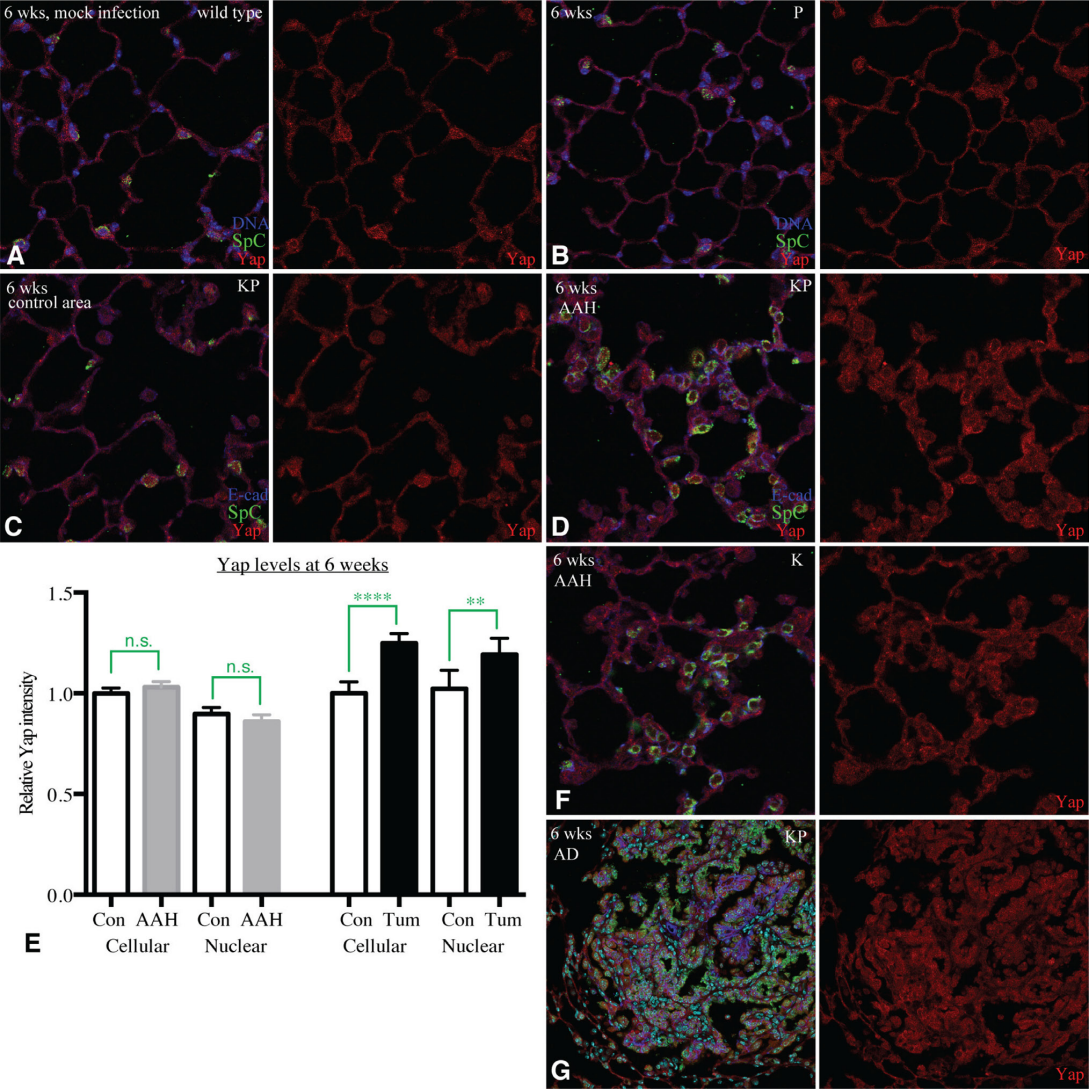
YAP is not upregulated by Kras and p53 (**A**–**D**) Representative images of lung sections from KP mice stained for Yap (red), SpC (green) and DNA or E-cad (blue). (A) Section from wild-type mouse, 6 weeks after mock infection. (B) Section from *P* mouse (negative control), 6 weeks after infection. (C) Section from KP mouse in an area without hyperplasias or tumors, 6 weeks after infection. (D) Section from KP mouse in an area with hyperplasia, 6 weeks after infection. (**E**) Histogram of YAP levels in hyperplasias (AAH) and control regions, and in a tumor and control regions, in KP mice 6 weeks after infection. Error bars show 95% confidence interval, statistical comparisons made by unpaired *t*-test; n.s., not significant, ^**^P < 0.01, ^****^P < 0.0001. (**F**) Representative image of lung section from K mouse, 6 weeks after infection, stained for Yap (red), SpC (green) and E-cad (blue). (**G**) Image of a tumor in a KP mouse lung sections, 6 weeks after infection, stained for Yap (red), SpC (green), DNA (cyan) and E-cad (blue). Quantification of Yap levels in this tumor is shown in (E).

The essential requirement for Yap in Kras-induced tumorigenesis raises the question of how Yap expression and activity in lungs is affected by oncogenic Kras. This was examined by assessing Yap levels and localization in lung sections stained for Yap, E-cad, Sp-C and DNA. In normal lungs, or lungs of control mice infected with Adeno-Cre, Yap is visible in the cytoplasm of both aveolar type 1 and AT2 cells (Figure 3A, 3B). In AT2 cells, low levels of Yap are also consistently detected in the nucleus (Figure 3A, 3B), consistent with observations that Yap is active in AT2 cells [28].

To assess whether changes in Yap levels or localization occur during tumor initiation, we examined lungs in KP mice 6 weeks after infection with Adeno-Cre. Hyperplasias (AAH) and tumors at this stage are recognizable as clusters of cells expressing Sp-C. To eliminate variation in staining intensity due to differences in processing, we compared Yap levels in cells within AAH with Yap levels in isolated AT2 cells in other, nearby, morphologically normal regions of the same lung slice. Quantitation of both nuclear and total cellular Yap levels in 6 AAH, from 3 representative lung lobes of 2 different mice, with 10–20 cells scored per AAH and a similar number of control cells scored, revealed no significant difference between AAH and control regions (Figure 3C–3E). This indicates that activated-Kras stimulates proliferation of AT2 cells without significantly promoting Yap activity, and implies that the appearance of clusters of cells with elevated Yap in AAH simply reflects an increased number and density of AT2 cells, rather than an increase in Yap activity stimulated by activated-Kras. AAH examined in K mice (with wild-type p53) also have Yap levels that appear similar to those in control AT2 cells (Figure 3F).

### Yap expression becomes heterogeneous during tumor progression

**Figure 4:**
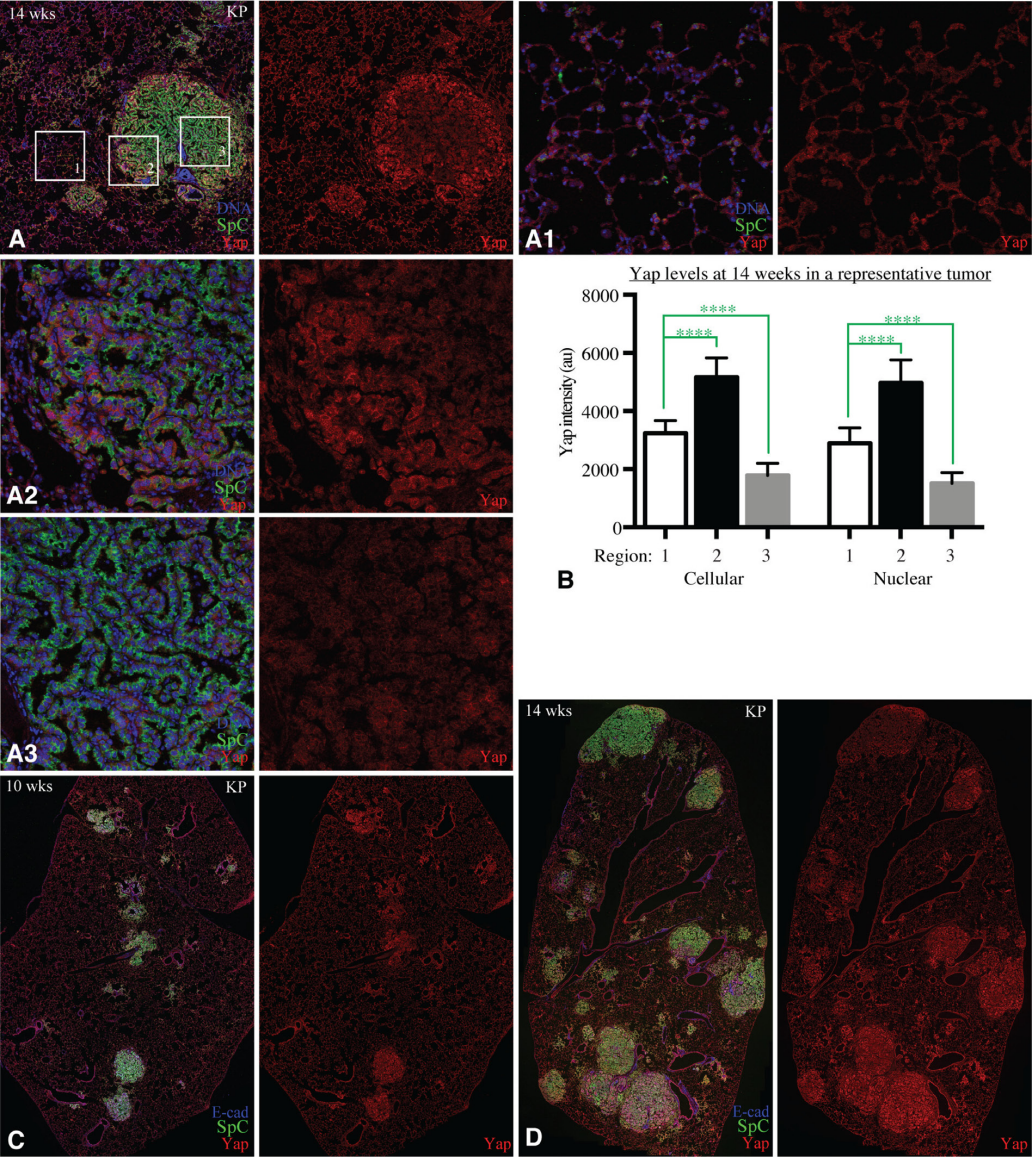
Yap expression becomes heterogeneous in tumors (**A**) Example of a tumor in KP mouse 14 weeks after infection, stained for Yap (red), SpC (green), and DNA (blue). Boxed regions are shown at higher magnification in (**A1**) (non-tumor control region), (**A2**) (tumor edge), and (**A3**) (tumor center). (**B**) Histogram showing quantitation of YAP levels in non-tumor AT2 cells (1), cells along tumor edge (2), and cells in tumor center (3). Error bars show s.d., significance of differences calculated by 1 way annova, ^****^indicates *P* < 0.0001. (**C**) Representative example of a lung section from KP mouse 10 weeks after infection, stained for Yap (red), SpC (green), and E-cad (blue). (**D**) Representative example of a lung section from KP mouse 14 weeks after infection, stained for Yap (red), SpC (green), and E-cad (blue).

To assess whether changes in Yap occur during tumor progression, we examined both nuclear and total cellular Yap levels in tumors of KP mice at 10 and 14 weeks after adeno-Cre infection. Examination of tumors at these later stages revealed substantial heterogeneity in Yap levels, both within and between tumors. In one common pattern, Yap levels in cells around the periphery of the tumor are higher than in cells near the center of the tumor (Figure 4A, 4B). Quantitation of relative Yap levels in tumors at 14 weeks after infection revealed that Yap levels near the edge of the tumor were either higher than (6/11 cases) or comparable to (5/11 cases) those in control ATII cells, whereas Yap levels in the central region of these tumors were usually lower than in control ATII cells (Figure 4A, 4B, 4D). Other tumors had heterogeneous levels of Yap that were not patterned from edge to center, and some tumors had relatively homogeneous Yap expression throughout the tumor, which could be higher, similar, or lower than in control AT2 cells (Figure 4D). Quantitation of relative Yap levels in tumors at 10 weeks after infection revealed a similar heterogeneity, with 4/9 tumors having regions with Yap levels elevated relative to control ATII cells, but cells in other regions of these same tumors had Yap levels at or below those in control ATII cells (Figure 4C). Intriguingly, we also identified a tumor in KP mice at 6 weeks after infection that had elevated levels of Yap (Figure 3E, 3G), by contrast to the lack of elevation in Yap levels in hyperplasias. These observations suggest that Yap levels correlate more with tumor progression than tumor genotype or age.

**Figure 5:**
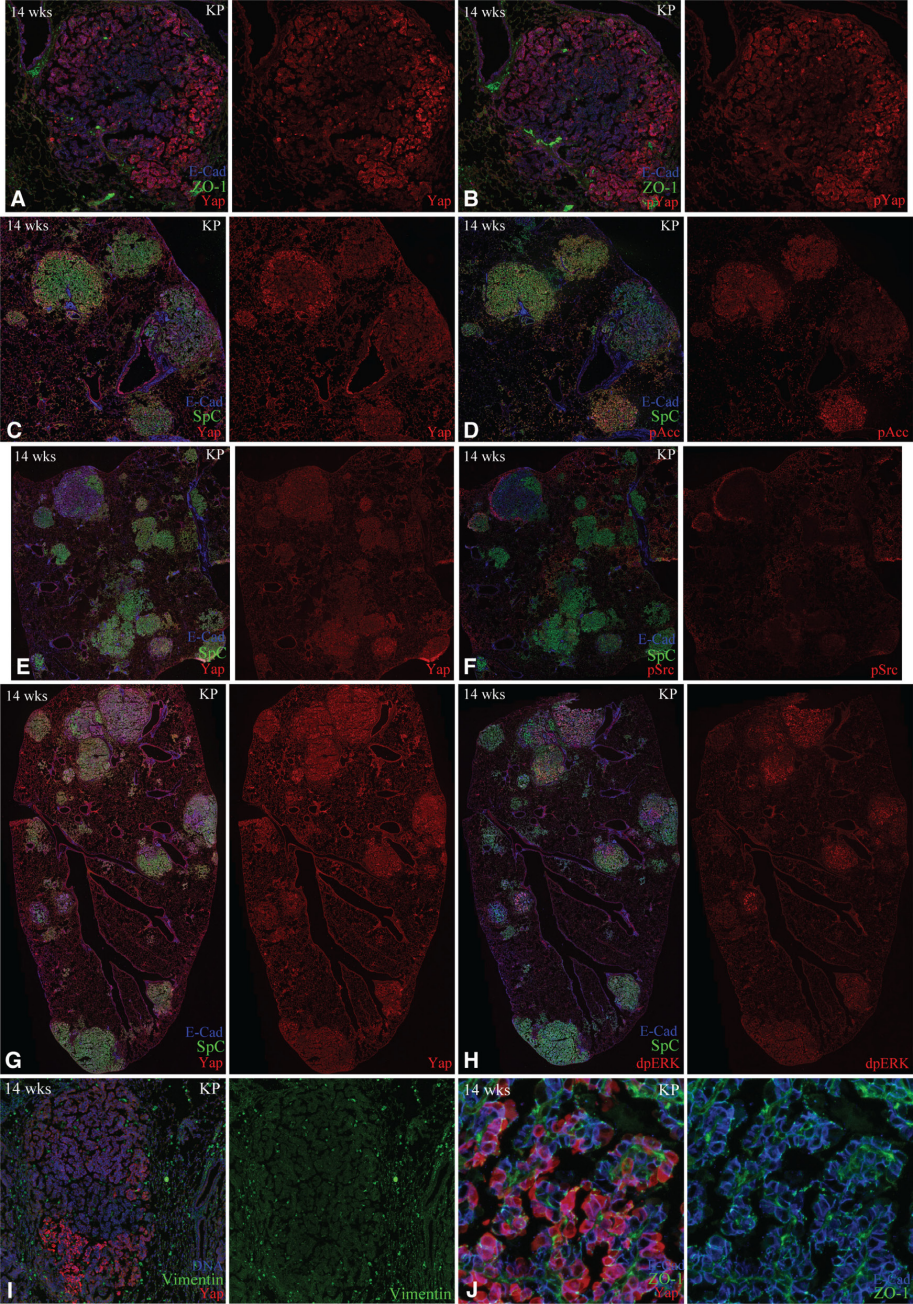
Yap heterogeneity is unrelated to markers examined All panels show examples of confocal stains of lung slices from KP mice, 14 weeks after infection. (**A**, **B**) Adjacent sections stained for E-cad, ZO-1, and either Yap (A), or phospho-Yap (B). (**C**, **D**) Adjacent sections stained for E-cad, SpC, and either Yap (C), or phospho-Acc (pAcc) (D). (**E**, **F**) Adjacent sections stained for E-cad, SpC, and either Yap (E), or phospho-Src (pSrc) (F). (**G**, **H**) Adjacent sections stained for E-cad, SpC, and either Yap (E), or diphospho-Erk (F). (**I**) Section stained for YAP (red), Vimentin (green), and DNA (blue). (**J**) Section stained for YAP (red), ZO-1 (green), and E-Cad (blue).

To gain insight into the variations in Yap levels, we used adjacent sections to compare Yap staining with markers that might be informative for Yap regulation. If variations in Yap levels stem from Hippo pathway-mediated regulation of Yap, then we would expect the pattern of phospho-Yap (S112) to be relatively high in regions where Yap staining is low, but instead they appeared to be quite similar (Figure 5A, 5B). In cases where Yap levels are relatively low within the center of the tumor, we considered the possibility that AMPK, which is activated at low cellular ATP to AMP ratios, and is known to down-regulate Yap [10], might be elevated in the center of large, compact tumors due to nutritional deficiencies. However, examination of levels of phosphorylation of Acetyl CoA carboxylase (ACC), which is a substrate of AMPK [35], did not reveal any difference between central tumor regions with lower Yap, and peripheral regions with higher Yap (Figure 5C, 5D). We also compared Yap staining to Src, which is known to regulate Yap, and to di-phospho-ERK, which is promoted by Kras activation, but did not observe consistent correlations between staining for these markers and Yap levels or localization (Figure 5E–5H). A subset of tumors had very high levels of Yap, and we investigated the possibility that this might be related to epithelial-mesenchymal transition (EMT), as EMT has been linked to Yap activation in some contexts [36]. However, Staining for E-cad, ZO-1, or Vimentin also did not reveal correlations with Yap levels or localization (Figure 5I, 5J).

### Yap levels correlate with tumor cell proliferation

**Figure 6:**
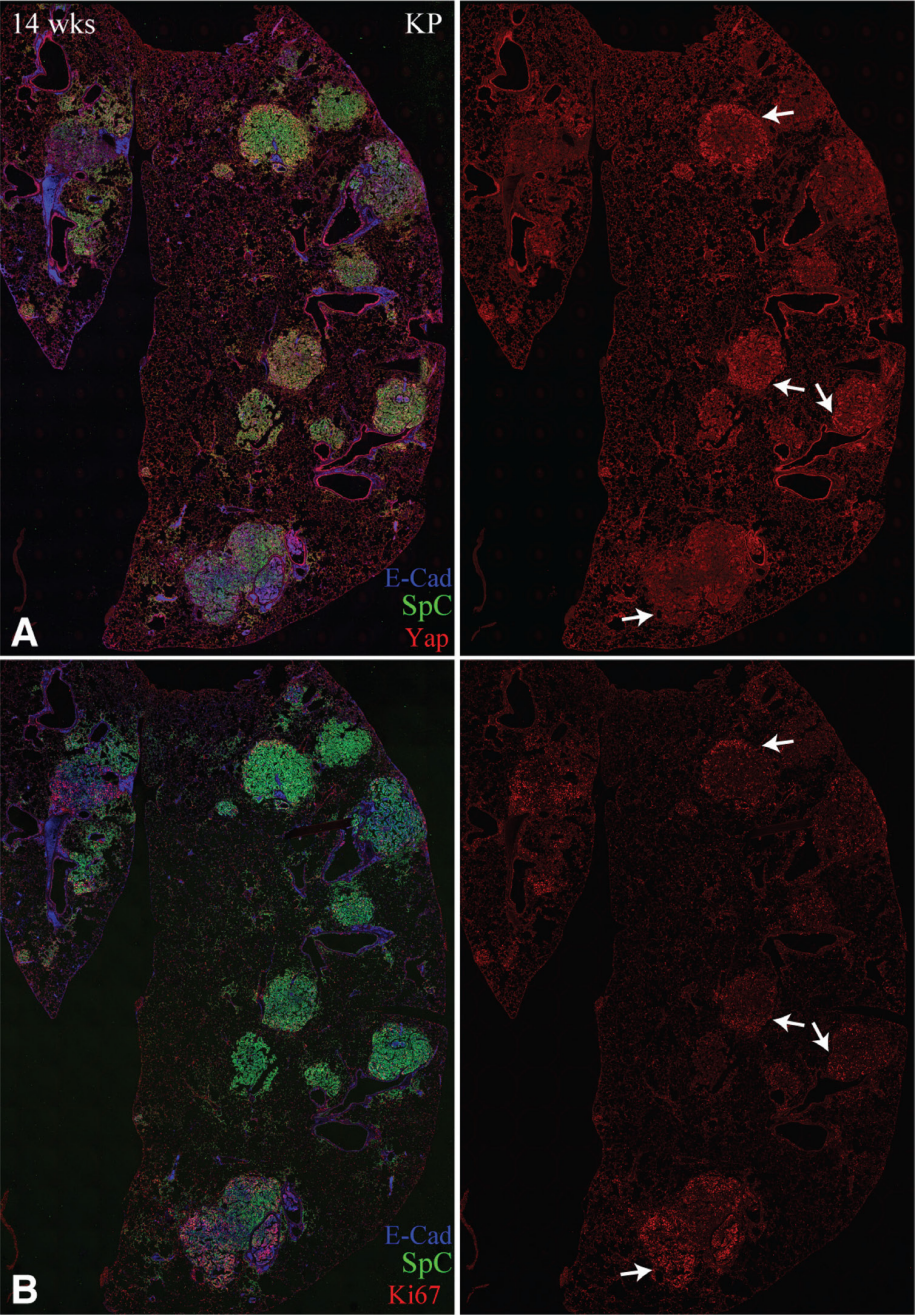
Yap levels correlate with Ki67 staining (**A**, **B**) Representative adjacent sections of lung slices from KP mice, 14 weeks after infection, stained for E-cad (blue), SpC (green), and either Yap (A), or Ki67 (B). Arrows point to examples of tumors with similar Yap and Ki67 staining.

Yap activation can promote growth and cell cycle progression. To investigate whether elevated Yap levels in Kras-induced tumors correlate with tumor cell proliferation, we compared Yap levels to staining for Ki67, which marks proliferating cells. In 40/45 tumors scored by immunofluorescence of adjacent sections, coming from 6 lobes of 2 different mice at 14 weeks after infection, Yap and Ki67 staining appeared correlated (Figure 6). This is most obvious in tumors that have a clear pattern of Yap staining, such as the tumors that have high Yap near the tumor periphery and low Yap in the tumor center. This correlation between Yap levels and cell proliferation is consistent with the hypothesis that Yap has a key functional role in promoting cell proliferation during lung tumorigenesis.

## DISCUSSION

Based on observations that Kras activation could increase Yap levels and nuclear localization in cultured cells, we had hypothesized that activation of Yap would be a key feature of Kras-induced lung adenocarcinoma. However, we instead found no evidence for elevation of Yap levels or nuclear localization during early stages of tumorigensis, as Yap levels in cells of AAH identified 6 weeks after induction of activated Kras were indistinguishable from those in control AT2 cells. Even in hyperplasias lacking p53, which has been noted to correlate with increased Yap levels and nuclear localization in Kras-induced tumors [23], Yap levels were not visibly elevated. Given evidence that AT2 cells are a cell of origin for lung adenocarcinoma [29–31], these observations suggest that Kras does not activate Yap directly in lung cells, but rather stimulates proliferation of AT2 cells.

As tumors progressed, we did observe increased Yap levels, which is indicative of Yap activation, but only in a subset of tumor cells. Moreover, in other tumor cells Yap levels actually become lower than in isolated AT2 cells. These observations imply that Yap levels are governed primarily by the local microenvironment of tumor cells, rather than the Kras or p53 genotype. Although we were not able to identify specific features of the tumor microenvironment that promote or inhibit Yap activation, given evidence for the key role of Yap in tumor progression [13, 14, 18, 23], and our observations correlating Yap levels in tumors with cell proliferation, defining these features of the microenvironment is an important goal for future studies.

Our experiments also revealed that Yap is absolutely essential to Kras-induced lung adenocarcinoma. Studies investigating the acquisition of resistance to suppression of Kras in Kras-induced tumors in pancreas, colon or lung identified Yap amplification and activation as a mechanism for maintenance of the tumor phenotype after extinction of activated-Kras but did not address the role of Yap activation during initiation of tumorigenesis [37, 38]. Yap was previously reported to contribute to Kras-induced lung adenocarcinoma, but it was suggested that this requirement was only partial [23, 24]. However, those experiments were done with partial knockdown by shRNA, or by Cre excision in which efficiency of excisions was not examined. By immunostaining for Yap, we established that no tumors ever form that do not express Yap.

Our observations also imply that Yap is not only required for initiation of tumorigenesis, but also has a key role in continued tumor growth, consistent with reports that YAP levels in human tumors correlate with tumor progression [13, 14, 18, 23]. Yap levels in tumors correlate with Ki67 labeling, which is a marker of cell proliferation. Moreover, we found that tumor size and progression were reduced in KPY mice as compared to KP mice. As all tumors were Yap-expressing, the only reasonable explanation for this observation is that heterozygosity for Yap suppresses tumor growth and progression. Dosage sensitivity of tumor growth to the *Drosophila* homolog of Yap, Yki, has been reported previously [39], and Yap heterozygosity can suppress liver overgrowth in mice mutant for the upstream Hippo pathway component NF2 [40]. This dosage sensitivity clearly supports Yap as a target for pharmacological treatments of lung adenocarcinoma, as it implies that even partial reduction of Yap activity could be beneficial to patients.

Some previous studies identified the Yap homolog Taz as contributing to lung adenocarcinoma [15–17]. We did not examine requirements for Taz as we found that elimination of Yap was sufficient to completely suppress tumorigenesis. We note that our results don’t exclude a contribution of Taz to Kras-induced lung adenocarcinoma, but they do indicate that in the absence of Yap, Taz alone is insufficient to support tumorigenesis.

## MATERIALS AND METHODS

### Mice and virus infection

Floxed *Yap* (*Yap*^*f/f*^) mice were provided by Duojia Pan (UT Southwestern). Kras ^G12D.LSL^ and floxed *Trp53* (*Trp53*^*f/f*^) mice were provided by Dr. Eileen White (CINJ, Rutgers University). Lox-stop-lox ZsGreen mice were purchased from The Jackson Laboratory (Cat. #: B6.Cg-Gt(ROSA)^26Sortm6(CAG-ZsGreen1)Hze^/J). Cre recombinase adenovirus Ad5CMVCre were purchased from the Viral Vector Core Facility of Iowa University (Cat#: VVC-U of Iowa-5). 6–8 weeks old mice were infected with Ad5CMVCre viruses by nostril inhalation at the dosage of 4 × 10^7^ PFU per mouse. The Ad5CMVCre virus stock was diluted with MEM medium and mixed with CaCl_2_ to final concentration of 10 mM before nostril inhalation. Mouse procedures were approved by the Institutional Animal Care and Use Committee (IACUC) at Rutgers University. For survival curves, male mice of each genotype were checked daily and sick mice were provided with gel-water and food on the cage bedding. Moribund mice were euthanized, and dead mice were autopsied for evidence of lung tumor growth and health status of other organs. Differences in survival were analyzed using GraphPad Prism software.

### Histology and microscopy

Mice were euthanized by cervical dislocation, and lungs were injected with 0.2–0.5ml 4% PFA via trachea depending on tumor burden, and the trachea was tied to prevent PFA from leaking. The lungs were then fixed in 4% PFA overnight at cold room, and processed to Optimal Cutting Temperature compound and frozen. 6–8 um sections were later cut using a Leica cryostat. Sections were processed for antigen retrieval in 10 mM sodium citrate (pH 6.0) at 95°C for 20–25 minutes, and incubated in primary antibodies overnight at 4 °C in PBS with 0.1% Triton X-100 and 1%BSA (PBSTB). Antibodies used included Yap1 (Cell Signaling Technology, #14074, 1:200), SP-C (Santa Cruz Biotechnology, #7706, 1:200-400), E-Cadherin (BD Biosciences, #610181, 1:200, and Invitrogen, #13-1900, 1:400), ZO-1 (Thermo fisher, #33-9100, 1:100-400), phospho-Yap (Cell Signaling Technology, #4911, 1:200), phospho-ACC (Cell Signaling Technology, #11818, 1:100), phospho-Src (R&D Systems, #AF2685, 10 ug/ml), Vimentin (Aves, #Vim, 1:400). and Phospho-p44/42 MAPK (Cell Signaling, # 4370, 1:200) Sections were then washed with PBSTB buffer or PBS buffer with 0.1% Tween 20 (PBST buffer), and then incubated with fluorescent secondary antibodies from Jackson Immunoresearch at room temperature for 2 hours. The sections were then washed with PBST or PBSTB buffer. Nuclear DNA was stained with Hoechst. Stained sections were imaged using a Leica SP8 confocal microscope. For whole lung lobe images, different areas were tile scanned at 20% overlap and then stitched together with mosaic merging in LAS X software.

For quantitation of Yap levels, mean Yap intensities of single cells (as outlined by E-cad staining) and nuclei (as defined by Hoechst staining) were quantified using Leica LAS X software. Measurements were made in a single Z plane where the nucleus of the selected cell appears largest. 10–20 cells were measured for each tumor region and nearby control regions.

For quantitation of tumor areas, tumors and hyperplasias were identified by SpC staining within stitched confocal scans of lung lobes, on 8 µm sections through the middle of each lobe, stained for SpC, E-cad, DNA, and Yap, and measured using Leica LAS X software. All five lung lobes of three animals were analyzed for each genotype at 6, 10, and 14 weeks post-infection. Statistical analysis was performed by one way anova using GraphPad Prism software.

For classification of tumor types, tumors and hyperplasias were identified by SpC staining within stitched confocal scans of lung lobes, on 8 µm sections through the middle of each lobe, stained for SpC, E-cad, DNA, and Yap. All five lung lobes of three animals were analyzed for each genotype at 6, 10, and 14 weeks post-infection and classified according to [33]. In atypical adenomatous hyperplasia (AAH), SpC positive cells proliferate continuously along the aveolar septae forming branched structures while maintaining normal alveoli organnization. In adenomas, SpC positive cells form clusters that occupy the whole alveoli. In adenocarcinomas, tumors develop heterogeneous SpC expression, irregular and enlarged nuclei, and irregular borders.

## Abbreviations

Aveolar type 2: AT2
Epidermal Growth Factor Receptor: EGFR
Atypical adenomatous hyperplasia: AAH
Surfactant protein C: SpC
